# Phylogeny of Microphthalminae Hartmann-Schröder, 1971, and revision of *Hesionella* Hartman, 1939, and *Struwela* Hartmann-Schröder, 1959 (Annelida, Errantia)

**DOI:** 10.7717/peerj.7723

**Published:** 2019-09-19

**Authors:** Sergio I. Salazar-Vallejo, Jesús Angel de León-González, Luis F. Carrera-Parra

**Affiliations:** 1 Departamento de Sistemática y Ecología Acuática, El Colegio de la Frontera Sur, Chetumal, Quintana Roo, México; 2 Laboratorio de Biosistemática, Facultad de Ciencias Biológicas, Universidad Autónoma de Nuevo León, San Nicolás de los Garza, Nuevo León, México

**Keywords:** Microphthalmids, Hesionidae, New genus, New species, Morphology

## Abstract

Microphthalminae Hartmann-Schröder, 1971 was proposed in Hesionidae to include *Microphthalmus* and *Hesionides*; however, the affinities of these genera to other members of Hesionidae have been debated, and some authors have concluded they do not belong in Hesionidae. Herein, based on morphological characters, a phylogenetic analysis of the subfamily and some other similar poorly-known genera, with an uncertain position in Hesionidae was performed to clarify their affinities. Our results indicate that Microphthalminae, as currently delimited, is paraphyletic. The inclusion of *Struwela*, *Uncopodarke*, and *Westheideius*, a new genus, as well as the recognition of *Fridericiella* are proposed to meet the requirement of monophyly; and as result of this, the elevation in rank to the family level is herein presented. Furthermore, the type species for *Hesionella* and *Struwela* are redescribed, and a new species in the latter is described. A key to identify microphthalmid genera is also included.

## Introduction

Hartmann-Schröder (1971:134) proposed Microphthalminae in her monograph of the German fauna. The diagnostic features were small body size (rarely longer than five mm), antennae, palps and cirri filiform, the former rarely set off from prostomium, parapodia biramous or subbiramous, and pygidium with anal lamellae and cirri. She only included in the subfamily the genera *Microphthalmus* Mecznikow, 1865 and *Hesionides* Friedrich, 1937 because some of their species were included in her monograph.

Fauchald (1977) did not follow the Hartmann-Schröder subfamilies, although he employed the subfamily category within five other families (Polynoidae, Sabellidae, Serpulidae, Syllidae, Terebellidae). Two other later publications avoided recognizing the subfamilies as well (Wilson, 2000; Rouse & Pleijel, 2001), although one of the compilations grouped *Hesionides* and *Microphthalmus* as Nereidiformia *incertae sedis*.

It has been stressed that *Hesionides* and *Microphthalmus* do not belong in Hesionidae (Pleijel, 1998; Pleijel & Dahlgren, 1998; Dahlgren et al., 2000; Pleijel & Gustavsson, 2010). Thus, it might be needed a new status for Hartmann-Schröder’s subfamily as indicated elsewhere (Salazar-Vallejo & Rizzo, 2009).

There are three other genera, traditionally included in Hesionidae which after Pleijel (1998) are regarded as *incertae sedis*: (a) *Alikunhia* Hartman, 1959 (replacement name for *Anophthalmus* Alikunhi, 1949; junior synonym of *Hesionides* after Westheide & Rao, 1977); (b) *Hesionella* Hartman, 1939 (close to *Microphthalmu*s fide Pleijel, 1998:*150*); and (c) *Struwela* Hartmann-Schröder, 1959 (uncertain affinity fide Pleijel, 1998:*91, 151*).

Two other genera might be related to the above three: (1) *Fridericiella* Hartmann-Schröder, 1959 (replacement name for *Hesionella* Friedrich, 1956); it was considered similar to *Hesionides arenaria* by Laubier *(1967: 5)*, while Westheide (1977:*107, 2013:416*) and Pleijel (1998:*158*) regarded it as a junior synonym of *Microphthalmus*; and (2) *Uncopodarke Uchida* in Uchida, Lopéz & Sato, 2019; these same authors (Uchida, Lopéz & Sato, 2019:*84*) concluded that it is closely related to *Struwela* and *Hesionella*.

On the other hand, at least one species in *Microphthalmus*, *M. hamosus* Westheide, 1982, has been regarded as symbiotic with other invertebrates. This species was recorded living on a sipunculid, and has marked morphological modifications including the dorsal displacement of the first three chaetigers, presence of modified neurohooks in these chaetigers, and hypertrophied anal lamellae.

Because of the above considerations, the phylogenetic affinities between those genera and most of the known species in Microphthalminae must be assessed to clarify their affinities, and we herein present a phylogenetic analysis of the group. As a result, Microphthalminae is redefined and elevated to family rank, *Westheideius* n. gen. is proposed for *Microphthalmus hamosus* because it markedly differs from other species in the genus, and *Fridericiella* is regarded as a valid genus-group name. Further, the type species for *Hesionella* and *Struwela* are redescribed, and a new species in the latter is described.

## Materials & Methods

### Phylogeny

The source of information was based on the original description and from recent redescriptions (Table 1).

**Table 1:** List of taxa used in the phylogenetic analysis and source of taxonomic information (OD, original description; RE, redescription).

Species	Source
*Nereimyra punctata* (Müller, 1788)	RE: Pleijel, 1998; Pleijel, Rouse & Nygren, 2012
*Oxydromus flexuosus* (delle Chiaje, 1827)	RE: Pleijel, 1998
*Sigambra phuketensis*Licher & Westheide, 1997	OD
*Fridericiella pacifica* (Friedrich, 1956)	OD
*Hesionella maccullochae*Hartman, 1939	OD, RE: This study
*Hesionides arenaria*Friedrich, 1937	OD
*H. bengalensis*Westheide, 1992	OD
*H. incisa*Yamanishi, 1984	OD
*H. indooceanica*Westheide & Rao, 1977	OD
*H. maxima*Westheide, 1967	OD
*H. minima*Westheide & Rao, 1977	OD
*H. peculiaris*Westheide & Rao, 1977	OD
*H. riegerorum*Westheide, 1979	OD
*H. unilamellata*Westheide, 1974	OD
*Micropththalmus aberrans* (Webster & Benedict, 1887)	OD, RE: Riser, 2000
*M. aggregatus*Riser, 2000	OD
*M. ancistrosylliformis*Hartmann-Schröder, 1962a	OD
*M. antarcticus*Bick, 1998	OD
*M. arenarius*Westheide, 1973	OD
*M. coustalini*Fournier, 1991	OD
*M. ephippiophorus*Clausen, 1986	OD
*M. hamosus*Westheide, 1982	OD
*M. hartmanae*Westheide, 1977	OD
*M. hystrix*Fournier, 1991	OD
*M. indefatigatus*Westheide, 1974	OD
*M. itoi Uchida* in Uchida, Lopéz & Sato, 2019	OD Uchida, 2004; Uchida, Lopéz & Sato, 2019
*M. listensis*Westheide, 1967	OD
*M. mahensis*Westheide, 2013	OD
*M. monilicornis*Hartmann-Schröder, 1962b	OD
*M. onychophorus*Westheide, 1994	OD
*M. pseudaberrans*Campoy & Viéitez, 1982	OD
*M. riseri*Westheide, 1994	OD
*M. sczelkowii*Mecznikow, 1865	RE: Westheide, 1967
*M. simplicichaetosus*Westheide & Purschke, 1992	OD
*M. southerni*Westheide, 1967	OD
*Struwela noodti*Hartmann-Schröder, 1959	OD, RE: This study
*S. camposi n. sp.*	This study
*Uncopodarke intermedia Uchida* in Uchida, Lopéz & Sato, 2019	OD Uchida, 2004; Uchida, Lopéz & Sato, 2019

### Phylogenetic analysis

The phylogenetic analysis was performed using maximum parsimony in PAUP 4.0a165 (Swofford, 2002). A two-step heuristic search was employed (Larkin, Neff & Simpson, 2006). In the first step, 100,000 random addition replicates were executed (starting seed 1858174998), holding only the five best trees in each. In the second step, the trees in memory were used as starting trees and were swapped using the TBR method (Tree Bisection Reconnection). All subjects (“characters”) were equal weight, and multiple subject-predicate relations (“multistate characters”) were unordered. Inapplicable data are scored as “–”, and treated as equivocal in the analysis. Character changes were examined using ACCTRAN optimization. The resulting trees and the transformation series were analyzed in MacClade. The strict consensus tree was only obtained and used to summarize the full resolved clades obtained in the analysis.

### Outgroup

Westheide (*1977:104*) indicated that *Nereimyra* De Blainville, 1828 or *Oxydromus* Grube, 1855 (referred to as *Ophiodromus* Sars, 1862; see Villalobos-Guerrero & Harris, 2012) could be the sister groups of *Microphthalmus*. *Nereimyra* has *Nereis rosea* Fabricius, 1780, described from Greenland as its type species (junior synonym of *Nereis aphroditoides* Fabricius, 1780). *Nereimyra* has been recently revised (Pleijel, Rouse & Nygren, 2012) and besides some observations on juveniles (Pleijel, 1998:*120*), the early development has been documented (Schram & Haaland, 1984) for *N. punctata (Müller, 1788)* and this will be used as an outgroup. *Ophiodromus* has *O. vittatus Sars, 1862*, described from Norway as its type species, and although the genus has not been revised, its status has been modified and is now regarded as a junior synonym of *Oxydromus*, with *O. fasciatus* Grube, 1855 from the Adriatic Sea as its type species (Villalobos-Guerrero & Harris, 2012). The early development has been studied by Haaland & Schram (1983), although these authors identified their Oslofjord specimens as *Ophiodromus flexuosus* Delle Chiaje, 1827 described from the Mediterranean Sea, whereas they had probably studied *O. vittatus* instead. This explains why we are using, as an outgroup, *O. flexuosus* as illustrated by Pleijel (1998). It must be taken in consideration that these two genera are not closely allied nor basal in hesionid phylogenetic studies, but belonging into different subfamilies (Psamathinae Pleijel, 1998 and Ophiodrominae Pleijel, 1998) after Summers, Pleijel & Rouse (2015). A pilargid species was used as an additional outgroup: *Sigambra phuketensis* Licher & Westheide, 1997.

### Ingroup

Because our focus is in the groups currently assigned to Microphthalminae and two other genera which might be related, the ingroup contains several generic group names: *Fridericiella* Hartmann-Schröder, 1959, *Hesionella* Hartman, 1939, *Hesionides Friedrich, 1937*, *Microphthalmus* Mecznikow, 1865, *Struwela* Hartmann-Schröder, 1959, and *Uncopodarke Uchida in* Uchida, Lopéz & Sato, 2019. Table 1 includes all species used in the analysis.

*Fridericiella* is a monotypic genus with *F. pacifica* (Friedrich, 1956, as *Hesionella pacifica* n. gen, n. sp.) from Lima, Peru living in gravel. Hartmann-Schröder (1959), in a footnote, proposed *Hesionella* as a replacement name for *Fridericiella* because it was pre-occupied by *Hesionella* Hartman, 1939. *Fridericiella* was considered similar to *Hesionides arenaria* by Laubier (1967), but it has also been regarded as a junior synonym of *Microphthalmus* by Westheide (1977) and Pleijel (1998).

*Hesionella* is a monotypic genus with *H. maccullochae Hartman, 1939* from California (see below for the new orthography); it was found living over the body of very long lumbrinerid polychaetes (*Lumbrineris zonata* Johnson, 1901). *Hesionella* has been regarded as “possibly near *Microphthalmus* Mecznikow, 1865” (Pleijel, 1998:*150, 159*). The only missing issue in the original description is that the holotype has large, compound falcate hooks in chaetiger 1, whereas other neurohooks have blades of varying size. The depressed body and the reduction of the dorsal cirri, together with the falcate hooks might be adaptations to an ectosymbiotic life.

*Hesionides* has *H. arenaria* Friedrich, 1937 from the North Sea as its type species. The genus has not been revised and there are about 13 species or subspecies recognized. Following the partial revision by Westheide (1967), Salazar-Vallejo & Rizzo (2009) grouped some species on the basis of the relative development of the anal plate, especially regarding the relative fusion of the lateral lobes, the shape of anal cirri, the number of notochaetae and of their teeth. Nine species were included in the phylogenetic analysis.

*Microphthalmus* is the largest genus in the group because it contains about 40 species; its type species is *M. sczelkowii* Mecznikow, 1865 from the North Sea. The genus has not been revised; Westheide (1967) made a key to 10 species grouped basically on the relative size of parapodial cirri, their number of notochaetae, and the type of neurochaetae. Salazar-Vallejo & Rizzo (2009), in their key to 10 tropical American species, used the type of noto- and neurochaetae, the pigmentation pattern, the presence of eyes, and the relative anal plate and anal cirri development. Herein, 21 species were included in the phylogenetic analysis.

*Struwela* was proposed for *S. noodti* Hartmann-Schröder, 1959 from El Salvador; it was found living over sand dollars (*Lanthonia longifissa* (Michelin, 1858)). The second species which is described herein (see below) as *S. camposi* n. sp. was found on two other sand dollar species (*L. grantii* Mortensen, 1948 and *Encope grandis* Agassiz, 1841) in the Northern Gulf of California. Three genera, *Struwela*, *Hesionella* and *Uncopodarke* have different neurochaetae in their first chaetiger and in the former, they are very large.

*Uncopodarke* has *U. intermedia Uchida* in Uchida, Lopéz & Sato, 2019 as its type and only species; it was described from Japan, but unlike *Hesionella* or *Struwela*, it was not found living on other marine invertebrate species. *Uncopodarke* resembles *Hesionella* because both have antennae placed over the anterior prostomial margin, palps minute, displaced ventrally, dorsal cirrophore expanded, and falcate compound neurohooks in first chaetiger. Their main difference is in the development of the anal plate because in *Uncopodarke* there are two anal cirri about as long as the fused anal membrane, whereas in *Hesionella* there are no anal cirri, and the anal membrane is separated into two lateral lobes.

### Excluded taxa

Two species were described upon posteriorly incomplete specimens: *Microphthalmus aciculata* Hartmann-Schröder, 1959, and *Microphthalmus sp C* Uebelacker, 1984. The description of *M. bermudensis* Westheide, 1973 was incomplete and with a few illustrations. *M. pettiboneae* Riser, 2000 was not included because its description was brief, without illustrations, and based upon specimens from both sides of the Atlantic.

*M. carolinensis Westheide & Rieger, 1987* together with *M. nahantensis* Westheide & Rieger, 1987, were described in a comparative approach and because several features were not detailed or illustrated, they were not included. Furthermore, four species, *M. bifurcatus Hartmann-Schröder, 1974*, *M. paraberrans* Hartmann-Schröder, 1982, *M. westheidei* Hartmann-Schröder, 1982, and *H. gohari* Hartmann-Schröder, 1960, were described based upon single and very small specimens; whereby, they were regarded as juveniles and excluded from the analysis.

*Microphthalmus sp A Uebelacker, 1984* is a presumed undescribed species which was well described and illustrated, but it was not included pending an evaluation of the available specimens for a future description. Five other species were removed because the original descriptions were not available, and they have not been redescribed: *M. biantennatus Wu, Zhao & Westheide, 1993*; *M. fragilis Bobretzky, 1870*; *M. similis Bobretzky, 1870*; *M. tyrrhenicus Zunarelli Vandini, 1967*; and *M. urofimbriata* Alikunhi, 1943.

Subspecies were regarded as very similar to their stem species; they were excluded on the assumption that they might not provide relevant information for evaluating the affinities among genera.

As indicated by Westheide (2013:*418*), *M. stocki* Hartmann-Schröder, 1980 does not belong in the genus, and if the neurochaetal handle is really chambered, it would not fall within Microphthalminae, because they have solid handles instead, whereas they are chambered in Hesionidae (Pleijel & Gustavsson, 2010).

### Characters

Characters were coded based on the “subject/predicate relationship” approach (Fitzhugh, 2006), which considers the subject as the character, and the predicate as the different states in which the character can be expressed. The data matrix includes 48 subjects, traditionally named characters (Tables 2 and 3), and it was edited in MacClade 4.08 (Madisson & Maddison, 2001).

**Table 2:** Characters, as subject-predicate relations used herein.

**Prostomium**	
1. Shape	0. Rectangular, sides parallel; 1. Trapezoidal, wider posteriorly; 2. Ovoid.
2. Posterior margin	0. Well defined; 1. Indistinct medially.
3. Antennae number	0. Three; 1. Two.
4. Antennae shape	0. Cylindrical, tapered; 1. Regularly constricted (moniliform).
5. Median antenna position	0. Anterior, over the anterior margin; 1. Central; 2. Posterior, towards the posterior margin.
6. Palps	0. Present; 1. Absent.
7. Palp articulation	0. Biarticulate; 1. Simple.
8. Palps position	0. Ventro-terminal (bases not visible from above); 1. Distal, on the anterior prostomial margin; 2. Ventral (palp bases not visible from above).
9. Eyes	0. Present; 1. Absent.
10. Number of eyes	0. Two pairs; 1. One pair.
**Anterior segments and cirri**	
11. Relative fusion between successive tentacular segments	0. Segments indistinct with cirri anteriorly displaced, not regularly separated; 1. Segments distinct with cirri regularly separated.
12. Pairs of cirri	0. Eight; 1. Six; 2. Four; 3. Three; 4. Two.
13. Size of cirri	0. Longer than body width; 1. As long as body width; 2. Shorter than body width.
14. Relative size of anterior cirri regarding dorsal cirri of chaetigers 1–2	0. Slightly longer or of about the same size; 1. Markedly longer (at least twice as long); 2. Shorter.
15. Cirri base	0. Cylindrical; 1. Subconical.
**Parapodia**	
16. Alignment	0. All neuropodia lateral; 1. Few anterior neuropodia dorsal; 2. First neuropodia ventral.
17. Dorsal cirri shape	0. Tapered; 1. Digitate; 2. Subdistally swollen.
18. Dorsal cirrostyle margins	0. Articulated; 1. Smooth.
19. Dorsal cirrophore	0. Cylindrical; 1. Subconical; 2. Globose or scale-shaped.
20. Dorsal cirri size	0. Three or more times longer than ventral cirri; 1. Twice as long as ventral cirri; 2. As long as ventral cirri, or shorter.
**Anterior parapodia chaetae**	
21. First chaetiger chaetae	0. Similar to chaetae in chaetiger 6; 1. Different from chaetiger 6.
22. Anterior neurohooks	0. Absent; 1. Present.
23. Anterior neurohook blade	0. Compressed, as long as wide; 1. Tapered, markedly longer than wide.
**Median parapodia notochaetae**	
24. Presence	0. Present; 1. Absent.
25. Number of notochaetae	0. 1–4; 1. five or more.
26. Notochaetae capillaries	0. Present; 1. Absent.
27. Notochaetae spines	0. Absent; 1. Present.
28. Notochaetae hooks	0. Absent; 1. Present.
29. Modified notochaetae denticulates	0. Absent; 1. Present.
30. Modified notochaetae pectinates	0. Absent; 1. Present.
**Median parapodia neurochaetae**	
31. Compound neurochaetae	0. Present; 1. Absent.
32. Handle	0. Chambered; 1. Solid.
33. Tips	0. Only bidentate; 1. Only unidentate; 2. Both uni- and bidentate.
34. Blades size in the same chaetiger	0. Heterogeneous, of markedly varying size; 1. Homogeneous, single-sized or of about the same size.
35. Neurochaetae spines	0. Absent; 1. Present.
36. Tips of spines	0. Only unidentate; 1. Only bidentate; 2. Both uni- and bidentate.
37. Denticulate neurochaetae	0. Absent; 1. Present.
38. Size in the same chaetiger	0. Heterogeneous, of markedly variable size; 1. Homogeneous, single-sized or of about the same size.
**Posterior end**	
39. Pygidium margin	0. Not projected into an anal membrane; 1. Transformed into an anal membrane.
40. Anal membrane lobes	0. Foliose; 1. Lobate; 2. Convoluted.
41. Anal membrane continuity	0. Continuous; 1. Medially notched; 2. Laterally separated or bipartite.
42. Anal membrane margin	0. Smooth; 1. Crenulated or fimbriated.
43. Anal cirri	0. Present; 1. Absent.
44. Shape of anal cirri	0. Tapered; 1. Basally swollen; 2. Medially or subdistally swollen.
45. Size of anal cirri	0. Two to five times longer than pygidial width; 1. As long as pygidium width; 2. Smaller than pygidium width.
**Male copulatory organs**	
46. Presence	0. Absent; 1. Present.
47. Position	0. On dorsal surface; 1. On prostomium; 2. Intersegmental.
**Habitat**	
48. Symbiotic life	0. Free-living; 1. Living in association with other invertebrates.

### Prostomium (1–10)

The outgroup has an ovoid, wider than long prostomium with smooth lateral contours; some ingroup taxa have a rather trapezoidal prostomium, usually wider posteriorly; in others it resembles a rectangle having parallel lateral margins. The prostomial posterior margin is visible and well-defined in the outgroup, but it can be obscured or ill-defined in some ingroup taxa. Most taxa have three antennae being mostly cylindrical, tapered, and a few ingroup taxa have moniliform antennae. The median antenna is present and placed anteriorly in the outgroup and some ingroup taxa, whereas it can be placed centrally or even towards the posterior margin in some other ingroup taxa. Palps are present in almost all taxa, but they are biarticulate in the outgroup, whereas they are simple in most of the ingroup taxa and one taxon, *Struwela*, lacks palps. Eyes are present in the outgroup and in many ingroup taxa, but they differ because in the outgroup there can be up to four eyes, whereas whenever they are present in the ingroup, they are minute and only two ones.

**Table 3:** Character matrix. Character numbers correspond to table 2 and cladogram in figure 1. ‘–’ denotes inapplicable data. *N. punctata*, *O. flexuosus*, and *S. phuketensis* were defined as outgroup.

	5 10 15 20 25 30 35 40 45
*N. punctata*	0 0 1 0 – 0 0 0 0 0 0 1 0 0 0 0 0 0 0 0 0 0 – 0 0 0 0 0 0 0 0 0 0 0 0 – 0 – 0 – – – 0 0 0 0 – 0
*O. flexuosus*	0 0 0 0 0 0 0 0 0 0 0 0 0 0 0 0 0 0 0 0 0 0 – 0 1 0 0 0 0 0 0 0 0 0 0 – 0 – 0 – – – 0 0 0 0 – 0
*S. phuketensis*	1 1 0 0 1 0 0 2 1 – 0 4 2 2 0 0 0 1 0 1 0 0 – 0 0 0 0 1 0 0 1 – – – 1 0 1 0 0 – – – 0 0 0 0 – 0
*F. pacifica*	1 0 0 0 2 0 1 2 0 1 1 1 1 0 0 0 0 1 0 1 0 0 – 1 – 1 0 0 0 0 0 1 0 0 0 – 0 – 1 0 0 0 0 2 0 0 – 0
*H. maccullochae*	1 1 1 0 – 0 1 2 1 – 0 1 2 1 0 2 1 1 0 2 1 1 0 1 – 1 0 0 0 1 0 1 1 0 0 – 0 – 1 0 1 0 1 – – 0 – 1
*H. arenaria*	1 1 0 0 2 0 1 2 1 – 1 3 1 1 0 0 1 1 0 1 0 0 – 0 0 1 0 0 1 0 0 1 0 0 0 – 0 – 1 1 2 0 0 0 0 0 – 0
*H. bengalensis*	1 1 0 0 2 0 1 2 1 – 1 3 1 1 1 0 0 1 1 0 0 0 – 0 0 1 0 0 1 0 0 1 0 0 0 – 0 – 1 0 0 0 0 0 0 0 – 0
*H. incisa*	2 1 0 0 2 0 1 2 1 – 1 3 0 1 0 0 0 1 0 1 0 0 – 0 0 1 0 0 1 0 0 1 0 0 0 – 0 – 1 0 1 0 0 0 0 0 – 0
*H. indoceanica*	2 1 0 0 2 0 1 2 1 – 1 3 0 1 1 0 0 1 1 1 0 0 – 0 0 1 0 0 1 0 0 1 0 0 0 – 0 – 1 0 0 0 0 0 0 0 – 0
*H. maxima*	1 1 0 0 2 0 1 1 1 – 1 3 0 1 1 0 0 1 1 1 0 0 – 0 0 1 0 0 1 0 0 1 0 0 0 – 0 – 1 0 1 0 0 0 0 1 0 0
*H. minima*	0 1 0 0 2 0 1 1 1 – 1 3 1 1 1 0 0 1 1 1 0 0 – 0 0 1 0 0 1 0 0 1 0 0 0 – 0 – 1 1 2 0 0 1 0 0 – 0
*H. pecularis*	0 1 0 0 2 0 1 1 1 – 1 3 0 1 1 0 0 1 1 1 0 0 – 0 0 1 0 0 1 0 0 1 0 0 0 – 0 – 1 1 2 0 1 – – 0 – 0
*H. riegerorum*	2 1 0 1 2 0 1 1 1 – 1 3 2 0 1 0 0 1 0 1 0 0 – 0 0 1 0 0 1 0 0 1 2 0 0 – 0 – 1 1 2 0 0 0 1 1 1 0
*H. unilamellata*	0 1 0 0 2 0 1 1 1 – 1 3 0 1 1 0 0 1 0 2 0 0 – 0 0 1 1 0 1 0 0 1 0 0 0 – 0 – 1 0 0 0 0 2 0 0 – 0
*M. aberrans*	2 0 0 0 2 0 1 2 0 1 1 1 0 1 0 0 0 1 0 2 0 0 – 0 1 0 0 0 0 1 0 1 0 0 1 1 0 1 1 0 0 1 0 0 0 1 2 0
*M. aggregatus*	2 0 0 0 2 0 1 2 0 1 1 1 0 1 1 0 1 1 0 0 0 0 – 0 0 1 0 0 0 1 0 1 0 0 1 2 0 0 1 0 0 1 0 0 0 1 2 0
*M. ancistrosyllisformis*	2 0 0 0 1 0 1 2 0 1 1 1 0 1 1 0 1 1 1 1 0 0 – 0 0 0 1 0 0 0 0 1 2 0 0 – 0 – 1 0 0 0 0 0 0 0 – 0
*M. antarcticus*	2 0 0 0 1 0 1 2 0 1 1 1 2 1 1 0 0 1 1 1 0 0 – 0 0 0 1 0 0 1 0 1 2 0 0 – 0 – 1 0 0 0 0 1 1 1 0 0
*M. arenarius*	1 1 0 0 2 0 1 2 0 1 1 1 0 1 1 0 0 1 1 2 0 0 – 0 0 1 1 0 0 1 0 1 0 0 1 0 0 1 1 0 0 0 0 0 0 1 2 0
*M. coustalini*	2 0 0 0 2 0 1 2 0 1 1 1 2 0 1 0 0 1 1 1 0 0 – 0 0 1 1 0 0 1 0 1 0 0 0 – 0 – 1 0 0 0 0 1 2 0 – 0
*M. ephippiophorus*	2 1 0 0 2 0 1 2 1 – 1 1 0 1 1 0 0 1 1 2 0 0 – 0 0 1 1 0 0 1 0 1 1 0 0 – 1 0 1 0 1 0 0 0 0 1 0 0
*M. hamosus*	2 0 1 0 – 0 1 2 0 1 1 1 0 1 1 1 0 1 1 1 1 1 0 0 0 1 0 0 0 1 0 1 2 0 0 – 0 – 1 2 1 0 1 – – 0 – 1
*M. hartmanae*	2 0 0 0 1 0 1 2 0 1 1 1 1 1 1 0 0 1 1 1 0 0 – 0 1 1 1 0 0 0 0 1 0 1 1 1 1 1 1 0 0 1 0 1 0 0 – 0
*M. hystrix*	2 0 0 0 2 0 1 2 0 1 1 1 0 1 1 0 0 1 1 0 0 0 – 0 1 1 0 1 0 0 0 1 1 0 1 2 0 0 1 0 0 0 0 1 0 0 – 0
*M. indefstigstus*	2 0 0 0 2 0 1 2 0 1 1 1 0 2 1 0 0 1 1 0 0 0 – 0 1 1 1 1 0 1 0 1 2 0 1 1 0 1 1 0 0 1 0 1 0 0 – 0
*M. itoi*	1 1 0 0 2 0 1 2 1 – 1 1 1 0 1 0 0 1 1 1 0 0 – 0 1 1 1 0 0 1 0 1 2 0 1 2 0 0 1 0 0 1 0 0 1 0 – 0
*M. listensis*	0 0 0 0 2 0 1 2 0 1 1 1 0 2 1 0 0 1 1 2 0 0 – 0 0 1 1 0 0 1 0 1 1 0 0 – 0 – 1 0 0 0 0 1 0 1 2 0
*M. mahensis*	2 0 0 0 2 0 1 2 0 1 1 1 0 0 1 0 0 1 1 0 0 0 – 0 0 1 1 0 0 1 0 1 0 0 1 2 1 0 1 0 0 1 0 1 0 1 2 0
*M. monilicormis*	1 0 0 1 2 0 1 2 0 1 1 1 0 0 1 0 0 1 1 1 0 0 – 0 1 1 1 0 0 0 0 1 0 0 0 – 0 – 1 0 0 0 0 2 0 0 – 0
*M. onychophorus*	2 1 0 0 2 0 1 2 1 – 1 1 0 0 1 0 0 1 1 1 0 0 – 0 1 1 1 1 0 1 0 1 0 0 1 1 0 1 1 0 0 0 0 1 0 0 – 0
*M. pseudoaberrans*	1 1 0 0 2 0 1 2 0 1 1 1 0 1 1 0 0 1 1 0 0 0 – 0 0 1 1 0 0 1 0 1 2 0 0 – 0 – 1 0 0 0 0 0 0 0 – 0
*M. riser*	1 0 0 0 2 0 1 2 0 1 1 1 0 1 1 0 0 1 1 1 0 0 – 0 0 1 1 0 0 1 0 1 2 0 0 – 0 – 1 0 0 0 1 – – 1 2 0
*M. sczelkowii*	1 0 0 0 2 1 1 2 0 1 1 1 0 1 1 0 0 1 1 1 0 0 – 0 0 1 0 0 0 1 0 1 2 0 0 – 0 – 1 0 0 0 0 0 0 1 2 0
*M. simplicichaetosus*	1 1 0 0 2 0 1 2 1 – 1 1 2 0 1 0 1 1 1 2 0 0 – 0 1 1 1 0 0 0 1 – – – 1 2 1 0 1 0 0 1 0 1 2 1 0 0
*M. southerni*	2 0 0 0 2 0 1 2 0 1 1 1 0 1 1 0 0 1 1 1 0 0 – 0 0 1 0 0 0 1 0 1 0 0 0 – 0 – 1 0 0 0 0 0 0 0 – 0
*S. noodti*	2 0 1 1 – 1 – – 0 1 0 2 2 0 0 2 3 1 2 2 1 1 1 1 – 1 0 0 0 0 0 1 0 1 0 – 0 – 1 1 2 0 1 – – 0 – 1
*S. camposi* n. sp.	2 0 1 1 – 1 – – 0 1 0 2 2 0 0 2 3 1 1 2 1 1 1 1 – 1 0 0 0 0 0 1 0 0 0 – 0 – 1 1 2 1 0 1 2 0 – 1
*U. intermedia*	2 0 1 0 – 0 1 2 0 1 1 1 1 1 0 2 1 1 2 1 1 1 0 0 0 1 0 0 0 1 0 1 0 0 0 – 0 – 1 0 0 0 0 0 2 0 – 0

### Anterior segments and cirri (11–15)

In the outgroup the anterior segments are usually markedly displaced anteriorly, such that the cirri are not regularly separated and segments can be fused, not separated dorsally, whereas in the ingroup taxa segments are separated dorsally, or at least the cirri are regularly separated, not projected forward as in some of the outgroup taxa. The anterior cirri can be numerous, with eight or six pairs in the outgroup, rarely only two pairs, whereas there are six or four pairs in the ingroup taxa. Anterior cirri are longer than body width in the outgroup taxa, and in some ingroup taxa, but many ingroup taxa have cirri smaller than body width. Further, these anterior cirri are of about the same size than corresponding dorsal cirri of the subsequent first chaetigers in the outgroup, whereas they are markedly longer in the ingroup taxa. The dorsal cirri bases are cylindrical in the outgroup as in most ingroup taxa, but some ingroup taxa have subconical bases.

### Parapodia (16–20)

Parapodia are all lateral in almost all included taxa; only one species (*M. hamosus*) has a few anterior parapodia directed dorsally, and members of *Struwela* and *Uncopodarke* have the first neuropodia directed ventrally. Dorsal cirri are tapered in most taxa but a few of the ingroup taxa have digitate cirri. The dorsal cirrostyle is articulated in the outgroup and smooth in the ingroup taxa. The dorsal cirrophore is cylindrical in the outgroup taxa, whereas most ingroup taxa have it subconical and in two ingroup taxa, *Struwela* and *Uncopodarke*, they are globose or scale-shaped. In the outgroup, dorsal cirri are markedly longer than ventral cirri, being up to three times longer, whereas it is rather shorter in the ingroup taxa, being twice as long as the ventral cirri or of about the same size.

### Chaetae (21–38)

The outgroup and most of the ingroup taxa have similar chaetae along the body, and only three taxa (*Hesionella*, *Struwela* and *Uncopodarke*) have larger, falcate, compound hooks in their first chaetiger. Notochaetae are present in the outgroup and in several of the ingroup taxa, but they are missing in some of the ingroup taxa. Notochaetae are distinguished from emergent aciculae mostly because of the exposed portion they show, with aciculae barely exposed and notochaetae markedly longer and more exposed. Further, notochaetae have been separated as smooth or modified; the smooth ones include capillaries, spines and hooks, whereas the modified ones include two basic types: (a) denticulates if there are some denticles along the longer, exposed margin; and (b) pectinates if their teeth are placed along the cutting or shorter margin. Notochaetae are simple capillaries in the outgroup and in some of the ingroup taxa, but there are some different chaetae in some of the ingroup taxa being mostly denticulate spines, which have teeth along a single series over the long, external margin, not alternating as in some hesionid genera, or pectinate chaetae which have spines along the short or cutting edge; a few ingroup taxa have both pectinate and capillary chaetae, whereas a few others have thick, simple hooks, and another one with hooks. Most notochaetae have tapered bases and some ingroup taxa have pectinate chaetae basally swollen, but not all pectinates are widened medially. Neurochaetae are all compound in the outgroup, whereas they can be compound or simple in most of the ingroup taxa, and rarely all simple in one species (*Microphthalmus simplicichaetosus Westheide & Purschke, 1992*). Compound neurochaetae are bidentate in the outgroup and in many ingroup taxa, but they can be unidentate only, or there can be both, bidentate and unidentate; their relative size is heterogeneous in the outgroup and many ingroup taxa, or it can be homogeneous.

### Posterior end (39–45)

The pygidium margin is not modified into an anal membrane in the outgroup, whereas it is variably developed in the ingroup taxa. In the ingroup, the anal membrane lobes can be foliose if they are wider than long or about as long as wide, lobate if they are longer than wide, or convoluted if the membrane turns over itself. The anal membrane can be continuous over its posterior margin, notched if there is a slight depression, or completely separated laterally or bipartite; its margin can be smooth, crenulated, or fimbriated. In the outgroup and in most of the ingroup taxa, the posterior end has two anal cirri; they are usually tapered in the outgroup, but they are modified in the ingroup taxa as basally swollen, or being medially or subdistally swollen. Anal cirri are markedly longer than pygidium in the outgroup and in some ingroup taxa, but in some of the ingroup taxa they can be as long as pygidium or even smaller than it.

### Male copulatory organs (46–47)

There are no copulatory organs in the outgroup, and some ingroup taxa have them. They can be placed over parapodia, between successive segments, on the dorsal surface, or even in the prostomium.

### Symbiotic life (48)

Most of the taxa studied are free-living; a few species have been found living on other marine invertebrates such as lumbrinerid polychaetes (*Hesionella*), or irregular sea-urchins or sand-dollars (*Struwela*).

### Revisions

Type materials are deposited in the Allan Hancock Foundation Polychaete collection, now housed in the Natural History Museum of Los Angeles County (LACM), Zoological Museum and Institute, University of Hamburg (ZMH), the polychaete collection of the Laboratorio de Biosistemática (UANL), Facultad de Ciencias Biológicas, U.A.N.L., Monterrey, Mexico, and the Reference Collection of Benthos (ECOSUR) of El Colegio de la Frontera Sur, Chetumal, Mexico.

Additional specimens were collected from sand-dollars; they were collected in the Northern Gulf of California and the results of their symbionts are available elsewhere (Campos, De Campos & De León-González, 2009). An Olympus SZ61 stereomicroscope and an Olympus BX51 optical microscope equipped with differential interference contrast (DIC) and a drawing tube (Camera Lucida), were used for the revision of the type specimens. To illustrate the descriptions, we made a series of digital photographs, which were stacked by using HeliconFocus to improve the depth of field. Also, some specimens of the new species of *Struwela* were processed to be observed by SEM. They were dehydrated in a series of progressive concentrations of hexamethyldisilazane (HMDS). Once air-dried, they were mounted on aluminum stubs and coated with gold for observation using a JEOL JSM-6010Plus-LA scanning electron microscopy at the Scanning Electron Microscopy Laboratory (LMEB), in ECOSUR-Chetumal.

The electronic version of this article in Portable Document Format (PDF) will represent a published work according to the International Commission on Zoological Nomenclature (ICZN), and hence the new names contained in the electronic version are effectively published under that Code from the electronic edition alone. This published work and the nomenclatural acts it contains have been registered in ZooBank, the online registration system for the ICZN. The ZooBank LSIDs (Life Science Identifiers) can be resolved and the associated information viewed through any standard web browser by appending the LSID to the prefix http://zoobank.org/. The LSID for this publication is: urn:lsid:zoobank.org:pub:6DA69051-BDCF-4439-9F55-121F817D2348. The online version of this work is archived and available from the following digital repositories: PeerJ, PubMed Central and CLOCKSS.

**Figure 1:**
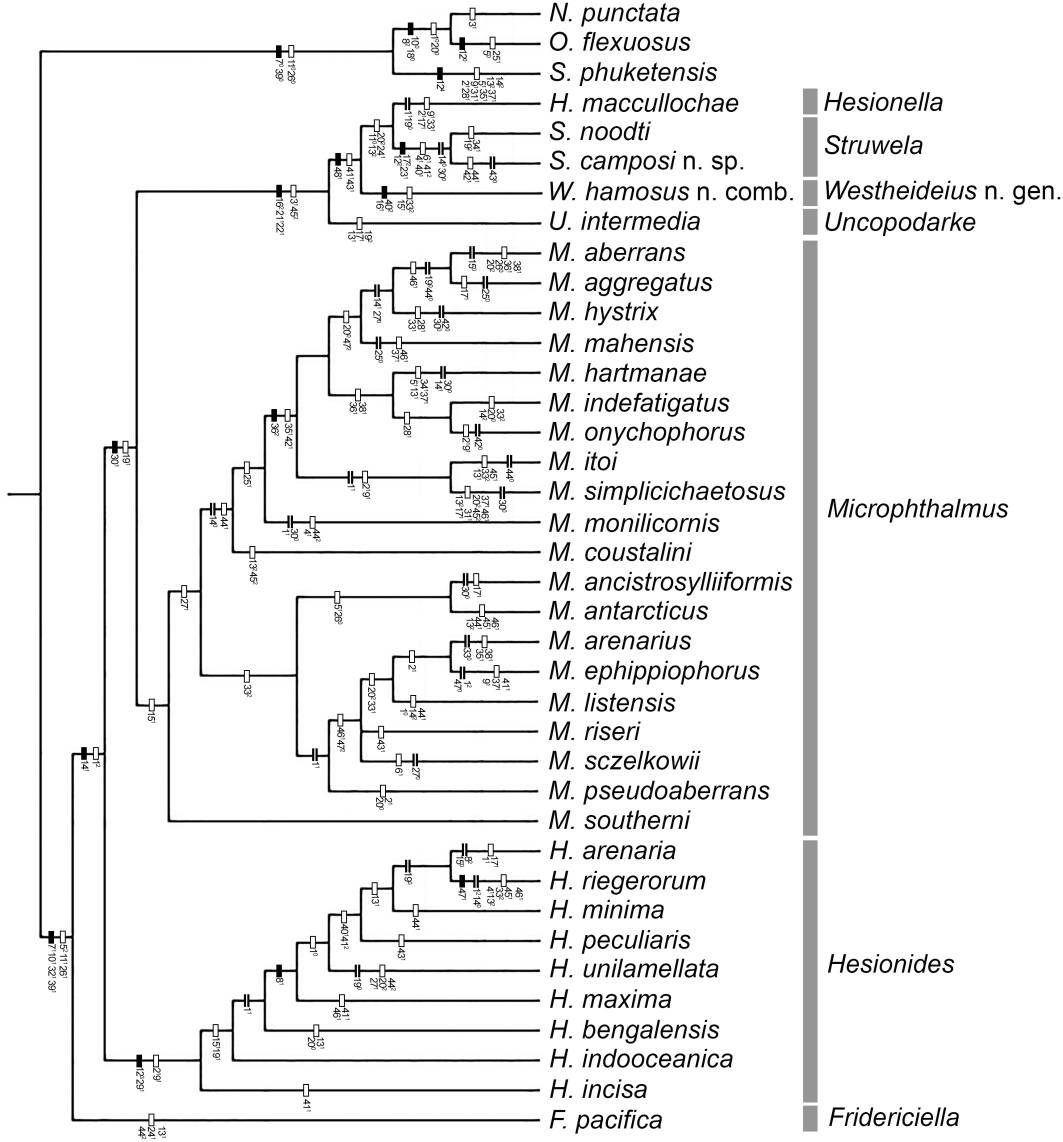
One of the three MPTs. Number in plain text are the characters and the superscript number is the character state occurring at the internode. Filled rectangle represent no homoplasy, blank rectangles represent homoplasy, double line represent reversal.

**Figure 2:**
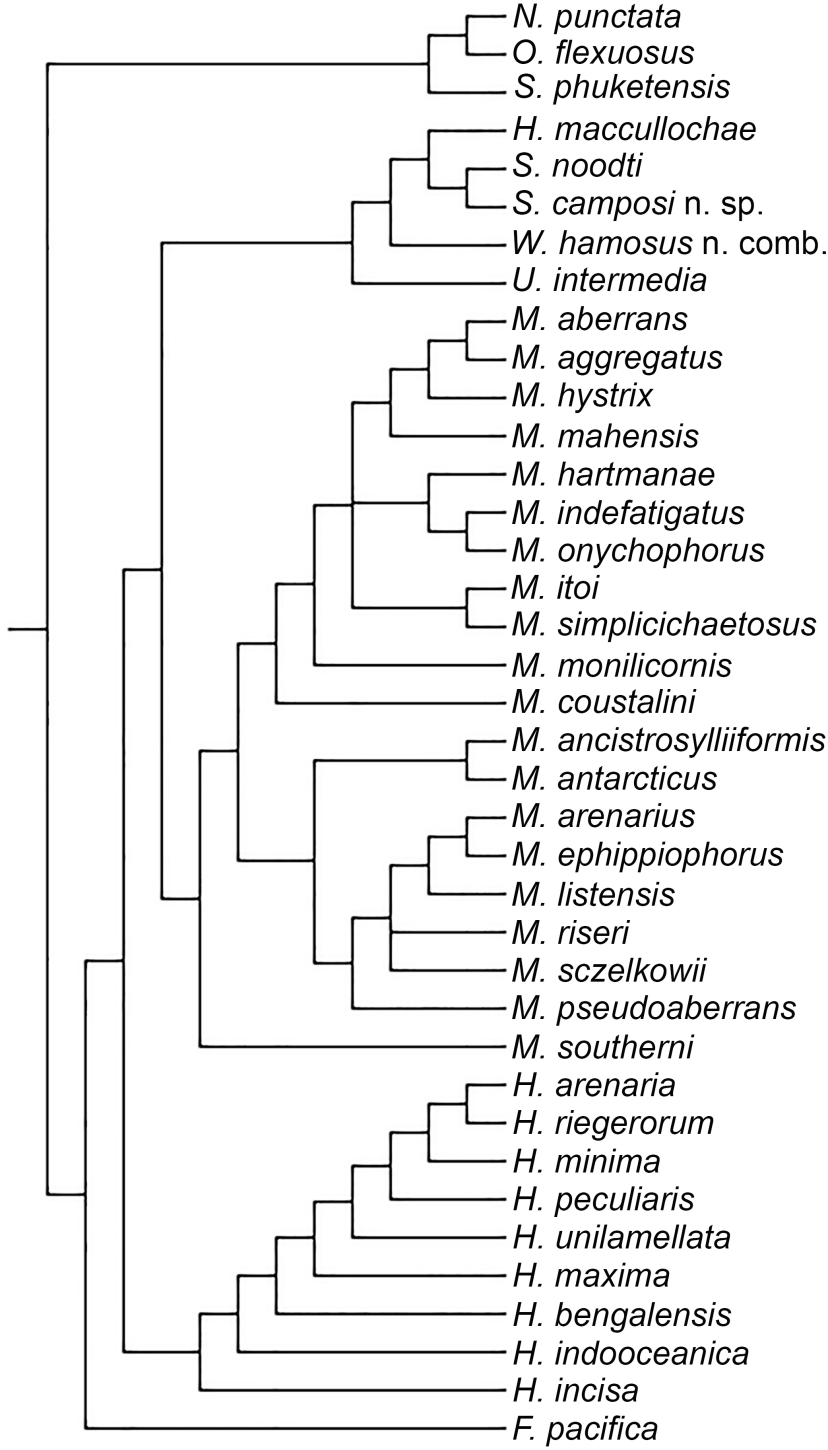
Strict consensus of three MPTs.

## Results

The analysis yielded three most parsimonious trees of 202 steeps, with a consistency index (CI) of 0.33, and a retention index (RI) of 0.58. One of the trees is shown to present the transformation series (Fig. 1); also, the strict consensus tree is shown in Fig. 2.

Our results show than Microphthalminae, as currently delimited, is paraphyletic. Herein, we propose the inclusion of *Struwela*, *Uncopodarke*, and *Westheideius* n. gen. and the recognition of *Fridericiella* as a valid genus to meet the requirement of its monophyly, and propose its elevation in rank to the family level. The monophyly of Microphthalmidae new status is characterized by the presence of pygidium transformed into an anal membrane, and neurochaetae with solid handle (not chambered), both non-homoplastic characters. Also, the family is recognized by the presence of simple palps (except in *Struwela* which lacks palps), anterior segments distinct with cirri regularly separated, except in *Hesionella* and *Struwela* which share anterior segments indistinct with cirri anteriorly displaced as the outgroup taxa, and the absence of capillary notochaetae, which are nevertheless present in three species of *Microphthalmus*. Each of these three character conditions represents reversal events into the ingroup clade. Herein, we define Microphthalmidae new status by including *Fridericiella*, *Hesionides*, *Microphthalmus*, *Uncopodarke*, *Westheideius* n. gen., *Struwela*, and *Hesionella*.

*Fridericiella* is the most basal taxon into the family; it is characterized by three homoplastic characters: (1) Anterior cirri as long as body width, a feature that occurs in five convergent events including *Uncopodarke*, two *Microphthalmus* species (*M. itoi* and *M. hartmanae*) and three species of *Hesionides* (*H. arenaria*, *H. bengalensis*, and *H. minima*). (2) Median parapodia without notochaetae, a feature shared with the clade *Hesionella*-*Struwela*. (3) Anal cirri medial or subdistally swollen, a condition shared with *Hesionides unilamellata* and *Microphthalmus monilicornis*. The taxonomic status of *Fridericiella* has been controversial, *Laubier (1967)* regarded it similar to *H. arenaria*, but it has also been regarded as a junior synonym of *Microphthalmus* by Westheide (1977, 2013) and by Pleijel (1998). However, our result shows that *Fridericiella* differs from all other Microphthalmid taxa. Therefore, the genus is herein reestablished and considered as a valid genus-group name belonging to Microphthalmidae.

The second clade corresponds to *Hesionides*, which is the sister taxa of the largest clade conformed by the other genera. It is characterized by having three pairs of anterior cirri and median parapodia with modified denticulated notochaetae, both non-homoplastic characters. Furthermore, it is characterized by two homoplastic characters: (1) Prostomium with posterior margin indistinct medially, a feature shared with one of the outgroup taxa (*Sigambra phuketensis*), *Hesionell*a, and six species of *Microphthalmus* (*M. onychophorus*, *M. itoi*, *M. simplicichaetosus*, *M. arenarius*, *M. ephippiophorus*, and *M. pseudoaberrans*). (2) Absence of eyes, shared also with *S. phuketensis*, *Hesionella*, *M. itoi*, *M. simplicichaetosus*, and *M. ephippiophorus*.

*Microphthalmus* is dived into two clades and with *M. southerni* as the basal species of the genus; however, *M. hamosus* is excluded to fulfill the monophyly of the genus (see below). This genus is defined by having anterior cirri with a subconical base; a homoplastic condition shared with some *Hesionides* species and *Westheideius* n. gen.

**Figure 3:**
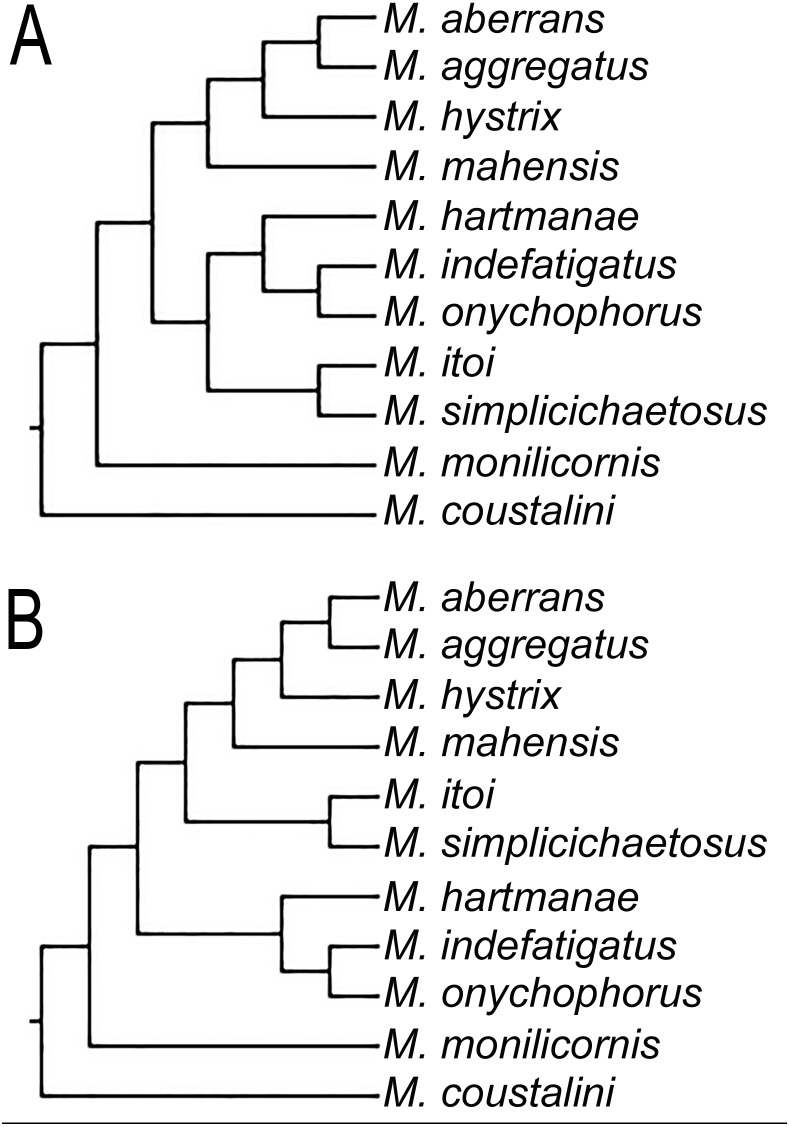
Different topologies found on the three MPTs for clade including some *Microphthalmus* species. (A) Clade *M. itoi*-*M. simplicichaetosus* as sister group of the clade *M. hartmanae*-(*M. onychophorus*-*M. indefatigatus*). (B) Clade *M. itoi* **-***M. simplicichaetosus* as sister group of the clade including *M. mahensis*-(*M. hystrix*-(*M. aggregatus*-*M. aberrans*)).

Even though our study was focused in the delineation of Microphthalmidae new status and their intrageneric relationships, our results show *Microphthalmus* mainly split into two clades, the largest including 11 species: *M. coustalini*, *M. monilicornis*, *M. simplicichaetosus*, *M. itoi*, *M. onychophorus*, *M. indefatigatus*, *M. hartmanae*, *M. mahensis*, *M. hystrix*, *M. aggregatus*, and *M. aberrans*. This clade is supported by having the anterior cirri slightly longer or of similar size than dorsal cirri of chaetigers 1–2 and anal cirri basally swollen, although both characters are variable within this clade. Likewise, the position of the clade *M. itoi*-*M. simplicichaetosus* was not resolved and two possible solutions were found, either as the sister group of the clade constituted by *M. hartmanae*-(*M. onychophorus*-*M. indefatigatus*) (Fig. 3A), or as the sister group of the clade which includes *M. mahensis*-*M. hystrix*-(*M. aggregatus*-*M. aberrans*) (Fig. 3B).

The second large clade of *Microphthalmus* is constituted by eight species and this clade is subdivided into two groups. The first one has two species: *M. ancistrosylliformis* and *M. antarcticus* both from the South Pacific (South of Chile and Antarctica). The remaining species: *M. arenarius*, *M. ephippiophorus*, *M. listensis*, *M. riseri*, *M. sczelkowii*, and *M. pseudaberrans* constitute the second group. All these species are from the Atlantic Ocean, except for *M. riseri* from New Zealand. The position of this latter species was not resolved, being part of the polytomy found in this clade, which was not resolved in any of the three most parsimonious trees of our analysis.

The sister clade of *Microphthalmus* is made up by four genera: *Uncopodarke*, *Westheideius* n. gen., *Hesionella*, and *Struwela*. It is supported by tree non-homoplastic characters: (1) First neuropodia ventral, except in *Westheideius* n. gen. which has first neuropodia in dorsal position, an autapomorphy into Microphthalmidae new status; (2) First chaetigers with chaetae different to those from chaetiger 6; (3) First chaetiger with neurohooks. Also, the clade is characterized by having two antennae, a feature shared with one of the members of the outgroup (*Nereimyra punctata*). *Uncopodarke*, as basal taxon of this clade, is characterized by having anterior cirri as long as body width, a feature shared with other taxa (see above, *Fridericiella* discussion); dorsal cirri digitate, a condition which appears in different convergent events and shared with *Hesionides arenaria*, *M. ancistrosylliformis*, *M. aggregatus*, and *Hesionella*; and by having globose dorsal cirrophores, a feature also present in *Struwela noodti*. *Uncopodarke* was proposed as belonging to Hesionidae by Uchida (2004, *nomen nudum* but fixed by Uchida, Lopéz & Sato, 2019). Its position into the family has not been elucidated in previous phylogenetic studies (Pleijel & Dahlgren, 1998; Pleijel, 1998; Ruta et al., 2007). Our results show that *Uncopodarke* does not belong in Hesionidae but in Microphthalmidae new status, closely related to *Westheideius* n. gen., *Hesionella*, and *Struwela*.

The clade including *Westheideius* n. gen., *Hesionella*, and *Struwela* is mainly supported by their symbiotic life, a non-homoplastic character. All of them have been found as symbiotic of other invertebrates as sand dollars, polychaetes or sipunculans. *Westheideius* n. gen. is characterized by having first anterior parapodia in dorsal position, and anal membrane lobes convoluted, both non-homoplastic characters. *W. hamosus* n. comb. was described as *Microphthalmus*, but our results do not support this proposed affinity. If this species is retained in *Microphthalmus*, it would derive into a paraphyletic group. Based on all the differences described above between *W. hamosus* n. comb. and *Microphthalmus* species, the viable solution is the proposal of an independent genus for this species. Herein, we propose *Westheideius* n. gen. (see below), including *M. hamosus* as its type species.

*Hesionella*, with its only species *H. maccullochae*, is characterized by having parapodia with cylindrical dorsal cirrophore, feature acquired in a reversal event and shared with *Fridericiella*, two *Microphthalmus* species (*M. aberrans* and *M. aggregatus*) and four species of *Hesionides* (*H. incisa*, *H. unilamellata*, *H. arenaria*, and *H. riegerorum*); it is also characterized by three other homoplastic features: (1) Prostomium with posterior margin indistinct medially, shared with other taxa (see above, discussion of *Hesionides*); (2) Presence of digitate dorsal cirri, shared with other taxa (see above, discussion of *Uncopodarke*); (3) Presence of only bidentate compound neurochaetae, a feature shared with three species of *Microphthalmus* (*M. hystrix*, *M. listensis*, and *M. ephippiophorus*).

*Struwela*, the sister taxon of *Hesionella*, is characterized by three non-homoplastic characters: (1) Presence of four pairs of anterior cirri; (2) Dorsal cirri subdistally swollen; (3) Anterior neurohooks with a tapered blade, markedly longer than wide. Furthermore, it is characterized by the absence of pectinate notochaetae, a condition acquired in a reversal event; antenna regularly constricted, a feature shared with *M. monilicornis* and *H. riegerorum*; the absence of palps, a condition also present in *M. sczelkowii*; and lobate anal membrane, laterally separated, conditions that appear in two independent events and shared with four species of *Hesionides* (*H. arenaria*, *H. riegerorum*. *H. minima*, and *H. peculiaris*).

*Struwela* was separated from other hesionid genera especially because of the lack of palps, and the presence of large, compound, falcate ventral hooks in chaetiger 1. There has been no other report on the species, besides the listing by Pleijel (1998:*151*) regarding it as a non-hesionid with uncertain affinities. Our result show *Struwela* is a member of Microphthalmidae new status based mainly in the presence of pygidium transformed into an anal membrane, neurochaetae with solid handle, and absence of capillary notochaetae. The absence of palps in *Struwela* is explained as a reversal event into the microphthalmids; variations about the development of palps are well known among polychaetes (Hesionidae, Pilargidae), from well-developed to missing, Thus, this morphological difference might not drive to the recognition of two different families for these genera.

## Systematics

**Table:** 

Phyllodocida Dales, 1962
Nereidiformia Glasby, 1993
Nereidoidea De Blainville, 1818

### Microphthalmidae Hartmann-Schröder, 1971 n. status

**Diagnosis emended**. Body small, delicate, rarely longer than five mm. Prostomium with 0–2 eyes. Antennae filiform. Palps filiform, sometimes missing. Tentacular cirri usually on 2–3 distinct segments. Dorsal cirri thin, smooth, thread-shaped. Parapodia biramous or subbiramous, lateral, rarely directed ventrally or dorsally. Pygidium transformed into an anal membrane, with angular or lobate anal cirri. No jaws. Free-living, rarely symbiotic with sand dollars, polychaetes or sipunculans.

**Table:** 

***Hesionella Hartman, 1939***
*Hesionella*Hartman, 1939: 159.

**Type species.** *Hesionella maccullochae Hartman, 1939*, by monotypy.

**Diagnosis.** Prostomium with one pair of divergent, lateral antenna, without median antenna. Palps minute, ventral. First three segments achaetous, each with paired dorsal and ventral cirri. Fourth segment first chaetiger; with reduced dorsal and ventral cirri, and large compound neurohooks with short blades. Following parapodia biramous. Dorsal cirri digitate. Notochaetae pectinates, fragile, singly per notopodium. Neurochaetae of two types, most chaetigers with compound falcigers, blades straight. Anal membrane with two lateral lappets.

**Table:** 

**Figure 4:**
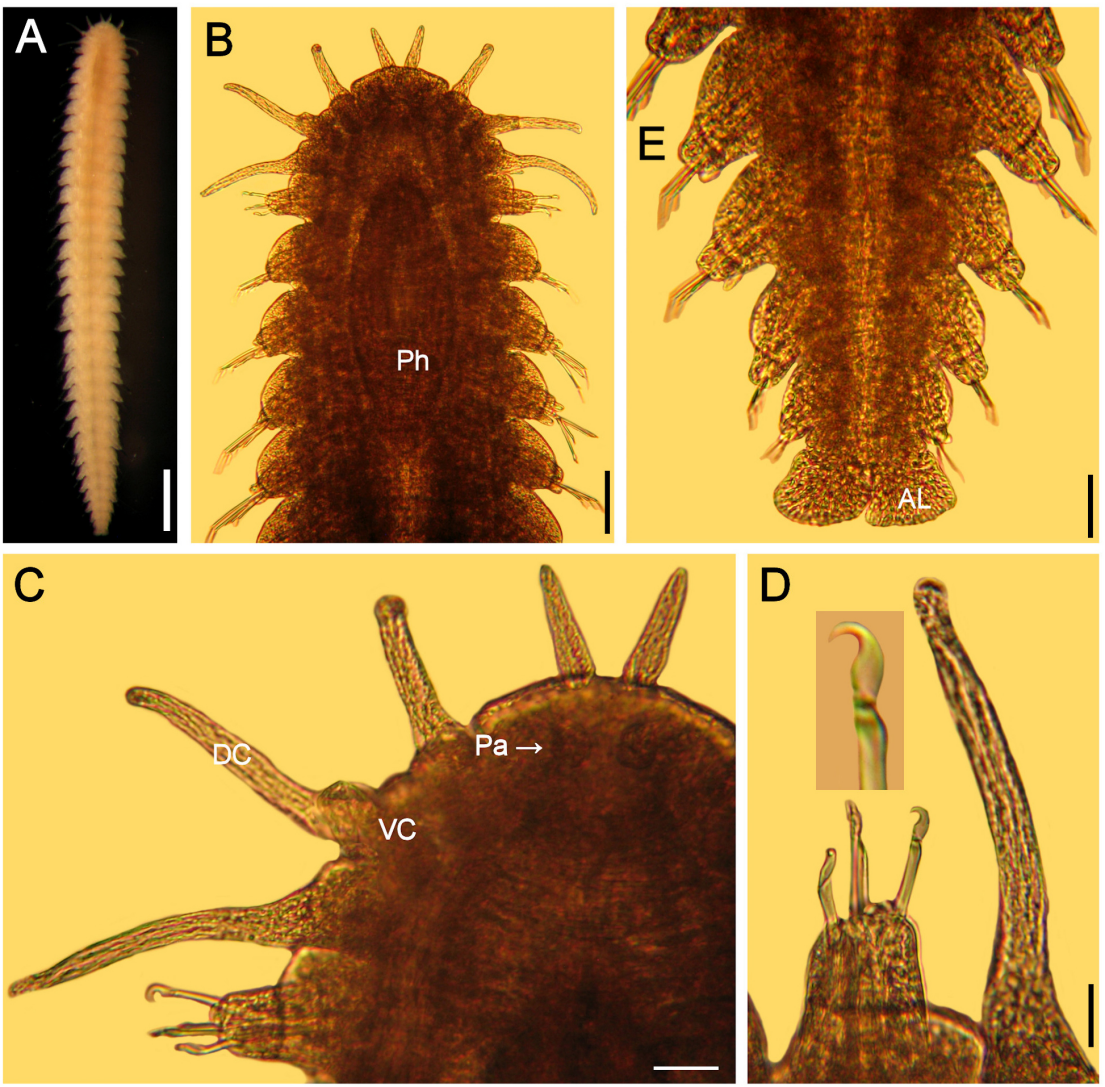
*Hesionella maccullochae*Hartman, 1939, holotype (LACM 129). (A) Ventral view. (B) Anterior region, ventral view (Ph: pharynx). (C) Close-up of same (DC: dorsal cirrus, Pa: palp, VC: ventral cirrus). (D) Chaetiger 1, left parapodium, close-up to show falcate, compound neurohooks (inset: blade). (E) Posterior region, ventral view (AL: anal lobe). Scale bars. A: 0.8 mm, B: 0.2 mm, C: 80 µm, D: 50 µm, E: 70 µm.

***Hesionella maccullochae Hartman, 1939***
Figure 4
*Hesionella mccullochae*Hartman, 1939: 159–161, Pl. 29, Figs 1–4; Blake & Walton, 1977: 313.

**Type material.** Northeasthern Pacific, California. Holotype (LACM 129), type locality after Hartman (1968:*367*), Newport Bay (33°36′31″N, 117°54′14″W), sand flats, intertidal, in burrows of *Lumbrineris zonata* (Johnson), date not specified.

**Additional material.** Northeasthern Pacific, California. One specimen (LACM 9326), Coal Point, Oregon, no further data (specimen bent ventrally, anterior cirri directed ventrally, wider than holotype; body 2.6 mm long, 0.5 mm wide, 27 chaetigers; falcate compound hooks in chaetiger 1; anal plate with two lateral, rounded lobes; median parapodia removed for photography).

**Redescription.** Holotype (LACM 129) complete; body blunt anteriorly, wider medially, tapered posteriorly (Fig. 4A), depressed (probably due to cover slip pressure), 6.5 mm long, 0.8 mm wide, 31 chaetigers

Prostomium short, anteriorly round, slightly projected, with two digitate, adjacent lateral antennae on its anterior margin, 4x longer than wide (Fig. 4B). Peristomium fused to prostomium, with two small rounded palps (about as long as wide) directed ventrally (Fig. 4C).

First three segments achaetous, with six pairs of tentacular (directed ventrally in LACM 9326); segments 1 and 3 with dorsal cirri cirriform, ventral cirri short, round (about as long as wide); segment 2 with both cirri cirriform, dorsal cirri 1/3 longer than ventral ones.

Following parapodia lateral throughout body, subbiramous (the original illustration seems to be based upon a mounted specimen because cirri are of similar length, but ventral one is shown longer). First chaetiger with reduced dorsal and ventral cirri. Notochaetae not seen, probably broken. Neurochaetae compound hooks, blades with short, falcate blade especially in chaetiger 1 (Fig. 4D, inset). Other parapodia with notopodial lobes depressed and larger neuropodial lobes; without notochaetae; neurohooks with straight blades, decreasing in size ventrally, tips falcate, unidentate, longest ones with blades 8×longer than wide.

Posterior region tapered; pygidium with anal lobes, foliose, blunt, expanded, margin slightly rugose (Fig. 4E); anal cirri not seen.

Pharynx seen by transparency, fusiform, thick, extended along chaetigers 1–5 (Fig. 4B). Oocytes not seen.

**Remarks.** In the original description of *Hesionella maccullochae Hartman (1939:160*) indicated 37–45 segments; it seems she had other specimens because she recorded 34–41 chaetigers, but the illustration corresponds with the holotype, which has 31 chaetigers only. Further, because two neurochaetae were illustrated, it is possible that some parapodia were removed from another specimen because the holotype has all parapodia on site.

The only missing issue in the original description is that the holotype has large, markedly falcate neurohooks in chaetiger 1. Other neurohooks have blades of varying size as originally indicated. The depressed body and the reduction of the dorsal cirri, together with the falcate hooks, might be explained by its adaptation to an ectosymbiotic life on lumbrinerids.

After the International Commission of Zoological Nomenclature, Formation of Names Guidelines (http://www.iczn.org/sites/iczn.org/files/Formation_of_names.pdf), section III, 21a, which literally indicates: The prefixes “Mac”, “Mc”, or “M”’ should be spelled “mac” and united, as in maccoki (McCook), maccoyi (M’Coy).” Consequently, the name should be modified to *Hesionella maccullochae*.

***Struwela*** **Hartmann-Schröder, 1959**

**Type species.** *Struwela noodti Hartmann-Schröder, 1959* by original designation.

**Diagnosis.** Prostomium with one pair of adjacent, frontal antennae, without median antenna. No Palps. First two segments achaetous, each with paired dorsal and ventral tentacular cirri. Third segment first chaetiger; with reduced dorsal and ventral cirri, and large compound neurohooks with long blades. Following parapodia sesquiramous. Dorsal cirri indistinctly articulated. No notochaetae. Neurochaetae of two type, most chaetigers with compound falcigers, blades straight. Anal membrane with two lateral lappets.

**Remarks.** *Struwela* differs from other microphthalmid genera in the absence of palps, by having four pairs of tentacular cirri, and by the presence of large, retractable compound neurohooks in chaetiger 1 (see discussion above, Phylogenetic section).

**Table:** 

**Figure 5:**
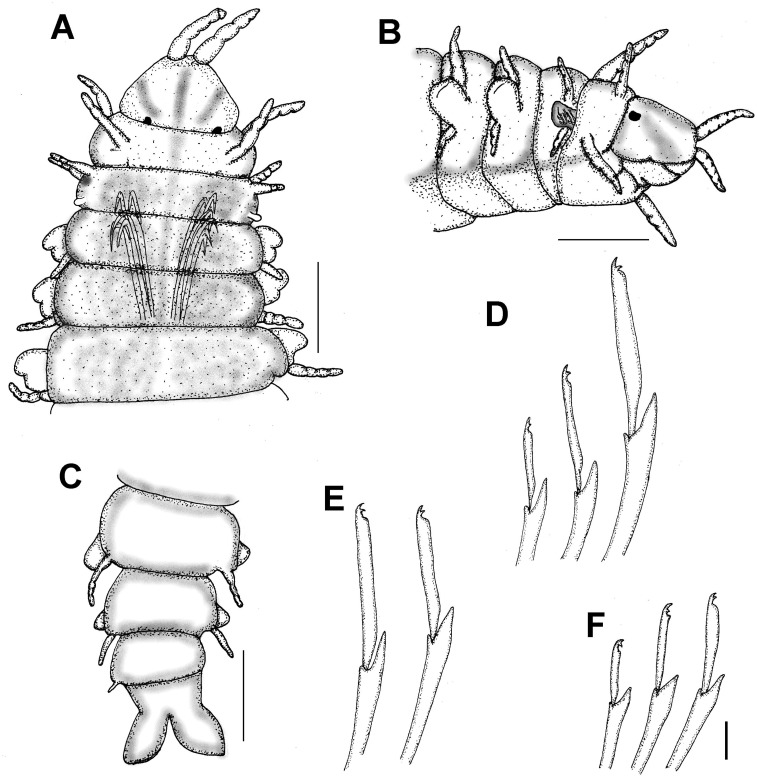
*Struwela camposi* n. sp., holotype (UANL 8126). (A) Anterior region, dorsal view. (B) Same, right lateral view. (C) Posterior region. (D–F) Neurochaetae from the same chaetiger. Scale bars. A: 0.1 mm, B–C: 0.2 mm, D–F: 15 µm. Drawing credit: Jesús Angel de León-González.

**Figure 6:**
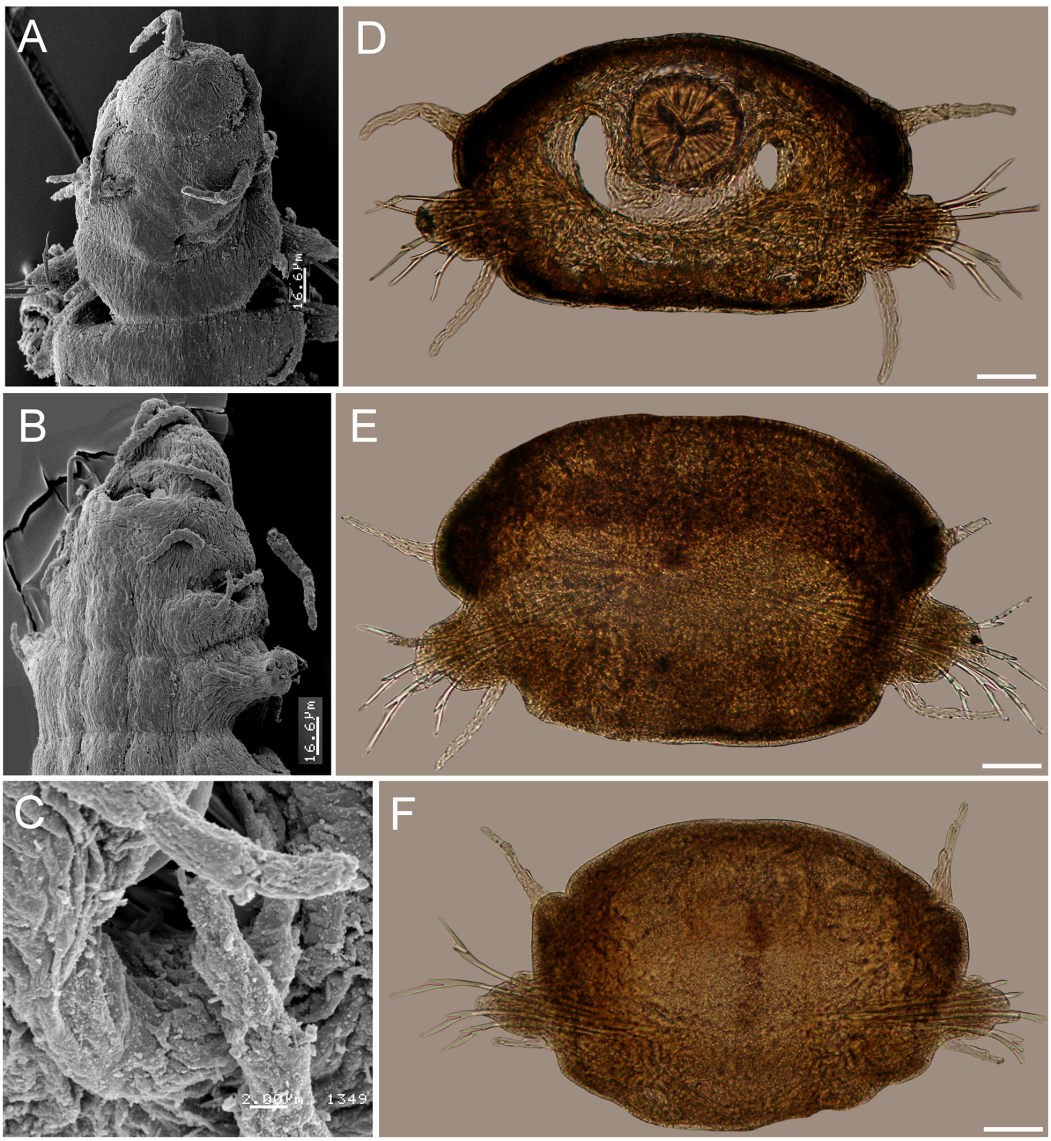
*Struwela camposi* n. sp., paratypes (UANL 8127). (A) Anterior end, dorsal view. (B) Anterior end, oblique ventrolateral view. (C) Same, cavity of modified anterior hooks, lateral view. (D) Cross section from chaetiger 11, pharynx with a Y-shaped lumen. (E) Cross section from chaetiger 30. (F) Cross section from chaetiger 55. Scale bars. A, B: 16.6 µm, C: 2 µm, D: 150 µm, E: 120 µm, F: 100 µm.

**Figure 7:**
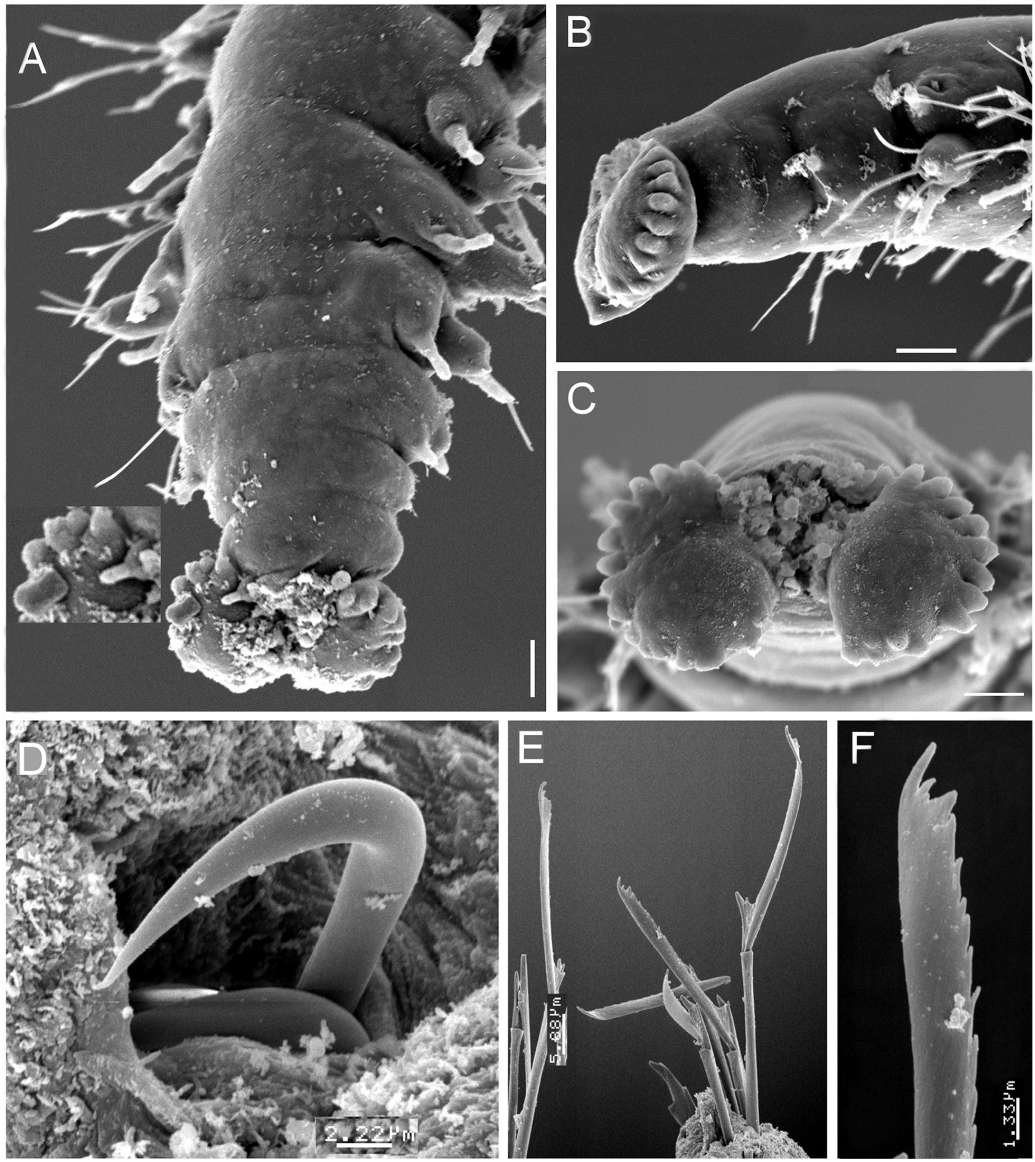
*Struwela camposi* n. sp., non-type specimens (ECOSUR). (A) Posterior region, dorsal view (inset: close-up of marginal papillae). (B) Another specimen, right lateral view. (C) Same, pygidium, posterior view. (D) Tip of modified hook from chaetiger 1. (E) Median chaetigers neurochaeteae showing different blade lengths. (F) Same, tip of neurochaetae. Scale bars. A, B: 20 µm, C: 15 µm, D: 2.22 µm, E: 5.88 µm, F: 1.33 µm.

**Figure 8:**
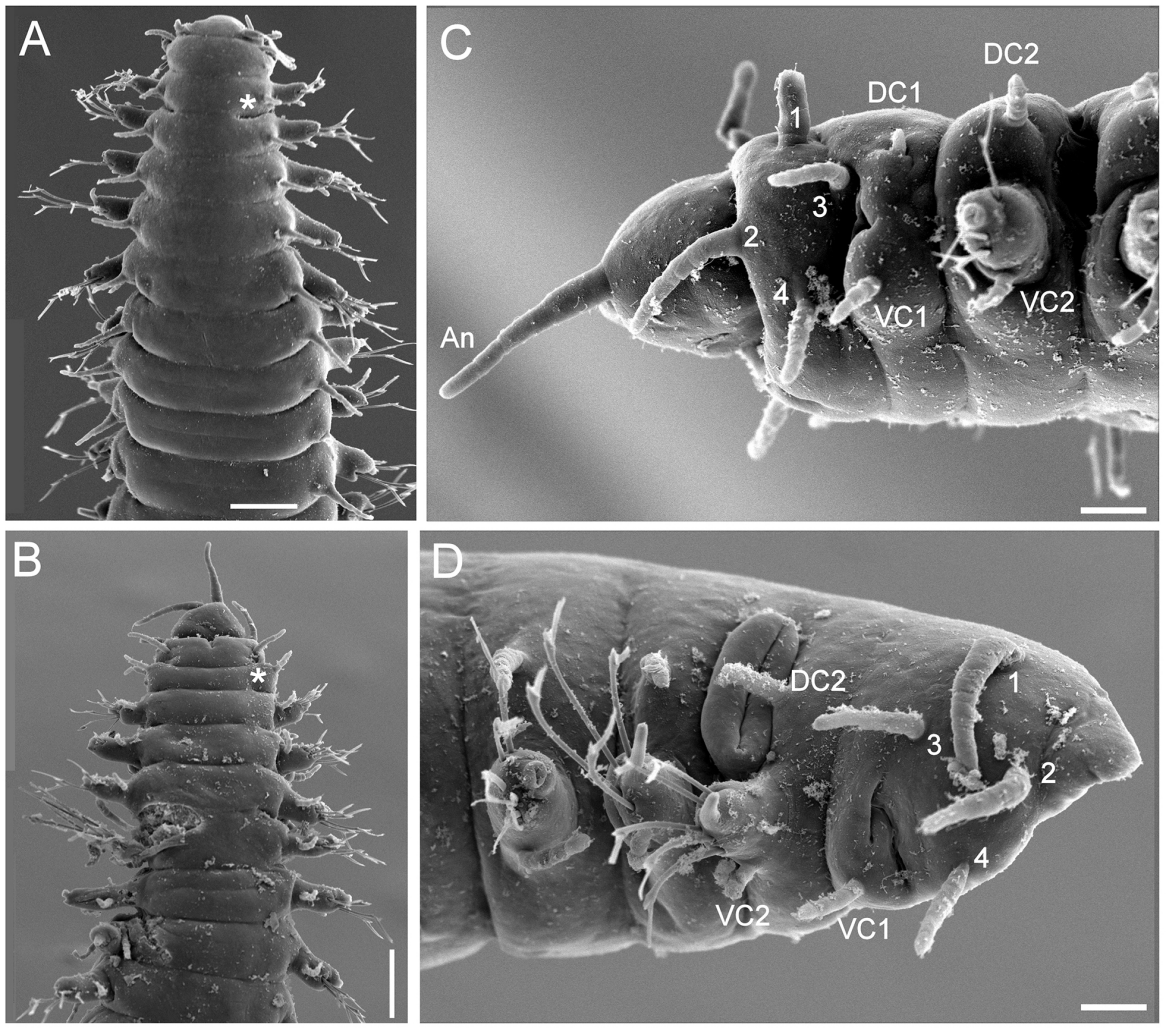
*Struwela camposi* n. sp., non-type specimens (ECOSUR). (A) Male, anterior region, dorsal view (asterisk indicates dorsal depression). (B) Male, anterior region, ventral view, antennae terminal (asterisk indicates cavity of modified anterior hooks). (C) Male, anterior region, left lateral view (numbers indicate tentacular cirri, An: antenna, DC: dorsal cirri, VC: ventral cirri). (D) Female, anterior region, right lateral view (numbers indicate tentacular cirri, DC: dorsal cirrus, VC: ventral cirrus). Scale bars. A: 140 µm, B: 120 µm, C–D: 20 µm.

***Struwela camposi*n. sp.**
urn:lsid:zoobank.org:act:76CAF1ED-BEBE-44E6-B2E1-3045D42C4DC4
Figs. 5–8
*Struwela* sp. Campos, De Campos & De León-González, 2009: 481–482, Fig 1.

**Type material.** Holotype (UANL 8126) and nine paratypes (UANL 8127), Rancho Punta Estrella, San Felipe, Baja California, Mexico, 31°04′11″N, 104°50′22″W, September 1995 on *Encope grandis Agassiz, 1841*, E. Campos, coll. Eight paratypes (ECOSUR 0213), same locality and collector, 27 September 2005, on *Lanthonia grantii (Mortensen, 1948)*.

**Additional material.** 20 specimens (UANL 8130), Campo el Pescador, San Felipe, Baja California, Mexico, 30°53′33″N, 114°51′09.3″W, 24 October 1988, on *L. grantii*; 41 specimens (UANL 0667), Rancho Punta Estrella, Baja California, 22 June 1994, E. Campos, coll.

**Description.** Holotype complete; body flat ventrally, first 17 segments with marked dorsal transverse dark brown bands, nine mm long, 0.8 mm wide, 69 chaetigers.

Prostomium subtriangular, wider than long, three dark frontal bands of pigment on anterior end, with two distal antennae longer than prostomial length, with four feebly defined articles. Eyes reniform, positioned towards prostomial posterior end, partially covered by the first segment anterior margin, laterally separated from it (Figs. 5A–5B).

First two segments achaetous, with four pairs of subequal tentacular cirri, each with four feebly defined articles; ventral cirri displaced forward, with three feebly defined articles each.

Parapodia sesquiramous. First chaetiger with two pairs of pseudo-articulated cirri formed by three incomplete articles, inserted laterally, closer to segmental anterior margin and one pair of papilliform cirri inserted on dorso-lateral posterior position, without neurochaetal lobe (Figs. 5A–5B, 8C–8D). Chaetiger 1 modified. A large, ventro-lateral depression in both sides (Figs. 6B, 8B); large retractile compound neurohooks emerge from it (Figs. 6C, 7D).

Neurochaetal lobes from chaetiger 2, lobate, blunt to tapered in posterior chaetigers. Dorsal and ventral cirri pseudoarticulated, subequal along a few anterior chaetigers, following chaetigers with ventral cirri slightly longer (Figs. 6D–6F), dorsal ones inserted in lateral projections, better defined in posterior chaetigers, slightly displaced dorsally in posterior chaetigers (Fig. 6F), ventral cirri inserted basally (Figs. 6D–6F), as long as dorsal cirri in anterior and median segments, progressively smaller in posterior chaetigers (Fig. 6F).

No notochaetae. Neurochaetae of two types. First chaetiger with long, curved compound neurohooks emerging from a latero-ventral depression (Figs. 6B–6C) in all specimens, these hooks appear inside the body in almost all specimens, hence retractable (Figs. 5A–5B). Compound bidentate falcigers with blades straight, of variable size, ventral one smaller, those of anterior and median chaetigers decreasing in size ventrally (Figs. 5D–5E), posterior falcigers with smaller blades (Fig. 5F). Some blades of anterior falcigers with spinulose inner margin (Figs. 7E–7F).

Posterior region tapered into a blunt pygidium, two preanal achaetous segments (Figs. 7A–7B); anal plate collar shaped, dorsally and ventrally divided into lateral lobes (Figs. 5C, 7C). Under higher magnification, anal plate lobate, smooth, margin crenulate, with 10–12 marginal short, blunt papillae; anal cirri minute, dorsolateral to anus (Fig. 7A, inset).

Pharynx muscular, tubular, extending between segments 8–15; in cross section, thin Y-shaped lumen (Fig. 6D). Oocytes not seen.

**Sexual dimorphism.** The dorsolateral surfaces just behind dorsal cirri of chaetiger 2 show two different modifications that are regarded as sexual dimorphism. Males have a deep depression (Figs. 8A–8C), without any additional surface features, but copulatory organs were not visible; if present, they can be retractable. Females with a massive external feature (Fig. 8D), with two thick, parallel fleshy outgrowths, and a longitudinal thin depression on it.

**Etymology**. This species name is after Ernesto Campos, a specialist of pinnotherid crabs and isopods from the Universidad de Baja California in Ensenada, in recognition of his friendship and support for our research, and especially because he collected the sand dollars with the specimens used for this description.

**Remarks.** *Struwela camposi* n. sp. differs from *S. noodti Hartmann-Schröder, 1959* mainly in the relative development of the falciger blades in the same chaetiger; in *S. camposi* anterior and middle segments have bidentate blades decreasing in size ventrally, and these segments do not have dorsal papillae, there are only two achaetous prepygidal segments, and this species lives on two sand dollar species, *Lanthonia grantii* and *Encope grandis*. On the contrary, *S. noodti* has falciger blades of similar size, median segments have isolated dorsal papillae, there are five achaetous prepygidal segments, and this species is known only as commensal of *L. longifissa (Michelin, 1858)*.

**Distribution.** This species is known from the northwestern part of the Gulf of California, near San Felipe Harbor, in the localities of Campo Pescador and Rancho Punta Estrella.

**Table:** 

**Figure 9:**
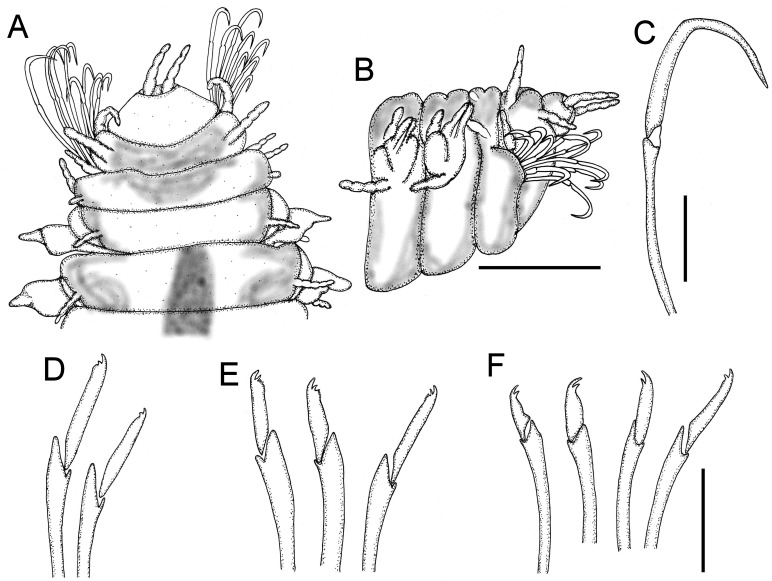
*Struwela noodti*Hartmann-Schröder, 1959, holotype (ZMH P-14195). (A) Anterior region, dorsal view. (B) Same, right lateral view. (C) Modified anterior hook, lateral view. (D–F) Neurochaetae showing slightly different blades. Scale bars. A, B: 0.1 mm, C: 25 µm, D–F: 15 µm. Drawing credit: Jesús Angel de León-González.

***Struwela noodti Hartmann-Schröder, 1959***
Figure 9
*Struwela noodti*Hartmann-Schröder, 1959: 107, Figs 41–46; Campos, De Campos & De León-González, 2009: 481–482, Fig. 2 (redrawn from the original).

**Type material.** Holotype (ZMH P-14195) and 22 Paratypes (ZMH P-14196), La Herradura beach, El Salvador, W. Noodt, Coll.

**Redescription.** Holotype complete; body flat ventrally, with dark brown (?) pigmentation throughout body, anteriorly with very dark pigmentation along segments two and three; holotype three mm long, 0.3 mm wide, without parapodia, with 40 segments; paratypes 3–4 mm long, 0.3–0.5 mm wide (without parapodia), up to 60 segments.

Prostomium subtriangular, wider than long, without pigmentation. Two distal antennae, slightly shorter than prostomial length, formed by four incomplete articles. Eyes not seen in any type specimen (Fig. 9A), probably faded out.

First two segments achaetous, with four pairs of tentacular cirri, dorsal ones bigger, inserted in middle lateral position, formed by five left and four right incomplete articles; anterolateral pair smaller, with four articles, ventrolateral pair formed by three articles.

Parapodia sesquiramous. First chaetiger as long as preceding segment, with two pairs of cirri, lateral ones with three incomplete articles, dorsolateral ones papiliform inserted on posterior margin of segment, without neurochaetal lobes. Chaetiger 1 modified, a ventro-lateral depression in both sides, from it emerges a group of modified compound hooks (Figs. 9A–9B). Other parapodia with lobate neurochaetal lobes. Dorsal and ventral cirri pseudoarticulated, those of first and second complete parapodia with ventral cirri longer.

No notochaetae. Neurochaetae of two types. First chaetiger with long and curved compound neurohooks, emergent between lateral part of first and second segments, apparently hooks are retractable (Fig. 9C). Other parapodia with compound falcigers with blades straight, of similar size, those of anterior and posterior part clearly bidentate (Figs. 9D–9F), those of middle body bidentate with a series of small denticles along cutting edge (Fig. 9E).

Posterior region tapered into a blunt pygidium, two preanal biannulate achaetous segments (or four single ringed achaetous segments); anal plate collar shaped, dorsally divided, not separated into divergent lobes.

Pharynx muscular, tubular, extending along segments four to thirteen.

**Remarks.** *Struwela noodti Hartmann-Schröder, 1959* was described with eyes but now they have faded out. As indicated above, it resembles *S. camposi* n. sp. but there are several important differences between them. The most important one is the relative development of the neurochaetal blades in the same chaetiger because in *S. noodti* they are of about the same size, whereas in *S. camposi* the same parapodium shows a ventrally decreasing range in their size. The other important difference is ecological because *S. noodti* was found on *Lanthonia longifissa*, whereas *S. camposi* was found on *L. grantii* and *Encope grandis*.

**Table:** 

***Westheideius*n. gen.**
urn:lsid:zoobank.org:act:1EF85063-0750-478A-814C-44E0E45A2DFA

**Diagnosis.** Body depressed. Prostomium with lateral antennae, no median antenna. Palps anteroventral. Six pairs of tentacular cirri. Anterior parapodia directed dorsally with compound falcigers with short, smooth, unidentate blades. Following chaetigers with compound falcigers with tapered, denticulate, uni- or bidentate blades. Anal plate with anal lamella convolute, turned on itself as an adhesive organ.

**Type species.** *Microphthalmus hamosus Westheide, 1982*.

**Etymology.** The genus-group is named after Prof. Dr. Wilfried Westheide in recognition of his contributions to the taxonomy of polychaetes in general, and especially because of his many studies on genera *Hesionides* and *Microphthalmus*, including the description of the type species for this newly proposed genus.

**Gender**. Masculine.

**Remarks.** *Westheideius* n. gen. resembles *Microphthalmus* but they can be separated easily because of several diagnostic features. *Westheideius* n. gen. has a depressed body, its anterior parapodia are directed dorsally, their neuropodia carry modified compound falcigers with short, smooth, unidentate blades, and its anal membrane is modified as a convoluted adhesive organ. In contrast, *Microphthalmus* has a rather cylindrical body, all parapodia are lateral and there are no modified compound falcigers, but rather have tapered, denticulate blades, and its anal membrane is foliose, never convolute. The type species, *M. hamosus Westheide, 1982* has been well described and illustrated by *Westheide (1982)* and *Uebelacker (1984)*.

### Key to genera of Microphthalmidae Hartmann-Schröder, 1971 new status

**Table:** 

1 With palps; tentacular cirri 3 or 6 pairs	2
– Without palps; tentacular cirri 4 pairs; first chaetiger with large compound neurohooks	*Struwela Hartmann-Schröder, 1959*
2(1) With 3 pairs of tentacular cirri; median parapodia with denticulate notochaetae	*Hesionides Friedrich, 1937*
– With 6 pairs of tentacular cirri; without denticulate notochaetae	3
3(2) Prostomium with median antenna; palps large	4
– Prostomium without median antenna; palps minute	6
4(3) All parapodia lateral; compound neurochaetae, if present, falcigers with blades tapered, longer than wide; anal membrane foliose, never convolute	5
– Anterior parapodia (chaetigers 1–5) dorsolateral; anterior compound neurochaetae with blades massive, as long as wide or slightly tapered; anal membrane convolute	*Westheideius* n. gen.
5(4) Median parapodia with notochaetae	*Microphthalmus*Mecznikow, 1865
– Median parapodia without notochaetae	*Fridericiella*Hartmann-Schröder, 1959
6(3) Pygidium with anal cirri, anal membrane continuous	*Uncopodarke Uchida* in Uchida, Lopéz & Sato, 2019
– Pygidium without anal cirri, anal membrane medially notched	*Hesionella*Hartman, 1939

## Discussion

From the original proposal by Mecznikow (1865:335) it was clear that the affinities of *Microphthalmus* with the hesionid genus *Podarke* were rather superficial, based upon the presence of three anterior achaetous segments, but differing by the type of chaetae, the anal plate, the pharynx papillae, and eyes. Friedrich (1937:345) proposed *Hesionides* and by the development of an anal plate, regarded it as similar to *Microphthalmus*, but with enough differences between them. Westheide (1967:13-14, 126) regarded these two genera as resembling more to each other than to the rest of the hesionid genera, because they have thin bodies, a reduction of sense organs, unarticulated cirri along body, and pygidium modified into an anal plate. He also pointed out that *Friedericiella* was also similar to *Hesionides* and *Microphthalmus*, and included a key to identify the latter two genera (Westheide, 1967:126), but he restrained himself for proposing a formal group for these genera.

It was Hartmann-Schröder (1971:126, 134) who proposed Micropththalminae as a new subfamily for *Microphthalmus* and *Hesionides*, and provided both a key to subfamilies, and diagnoses for the hesionid subfamilies. However, the other genera with a variably developed anal plate such as *Friedericiella*, or *Hesionella* were not included.

The proposal of the subfamily was based upon comparative morphology methods. After the study of some similar additional genera, such as *Hesionella*, *Struwela* and *Uncopodarke*, together with a cladistics analysis of the affinities between *Hesionides* and *Microphthalmus* species, we have corroborated that these genera conform a discrete group. Further, because this group has enough differences from Hesionidae, we have proceeded to propose an elevation in rank to the family level to Microphthalmidae Hartmann-Schröder, 1971. However, in order to avoid defining it as a paraphyletic group, we must recognize a new genus, *Westheideius*, different from *Microphthalmus*, such that Microphthalmidae includes *Friedericiella*, *Hesionella*, *Hesionides*, *Microphthalmus*, *Struwela*, *Uncopodarke* and *Westheideius*. At least *Hesionella*, *Struwela* and *Westheideius* were found as symbionts with other marine invertebrates, but the other genera include free living species.

## Conclusions

Microphthalminae was proposed for two genera and we evaluated if some other apparently related genera would match the diagnosis for the subfamily. We found that Microphthalminae, as currently defined, is paraphyletic. Based on our phylogenetic analysis, we propose the inclusion of *Struwela*, *Uncopodarke*, and *Westheideius* n. gen., as well as the recognition of *Fridericiella* to satisfy the requirement of monophyly. Consequently, we elevated it in rank to family level as Microphthalmidae new status. Now, the family includes seven genera: *Fridericiella*, *Hesionella*, *Hesionides*, *Microphthalmus*, *Struwela*, *Uncopodarke*, and *Westheideius* n. gen. Two genera (*Microphthalmus* and *Hesionides*) contain the higher number of species, all other genera are monospecific, except *Struwela* in which a second species was herein described. We consider that this low number of species is due to their cryptic lifestyle, three of the genera (*Westheideius* n. gen., *Hesionella*, and *Struwela*) are symbiotic with other invertebrates. We expect that as long as their potential hosts are reviewed for symbiotic polychaetes, the number of species will increase, as was shown here for *Struwela*.

## Additional Information and Declarations

### Data Availability

The following information was supplied regarding data availability: The specimens reviewed of *Hesionella maccullochae* are deposited in the Allan Hancock Foundation Polychaete collection, now housed in the Natural History Museum of Los Angeles County (LACM 129, LACM 9326). The specimens of *Struwela camposi* are deposited in the polychaete collection of the Laboratorio de Biosistemática, Facultad de Ciencias Biológicas, Monterrey, Mexico (UANL 8126, UANL 8127, UANL 8130, UANL 0667) and in the Reference Collection of Benthos of El Colegio de la Frontera Sur, Chetumal, Mexico (ECOSUR 0213). The specimens of *Struwela noodti* are deposited in the Zoological Museum and Institute, University of Hamburg (ZMH P-14195, ZMH P-14196).

### New Species Registration

The following information was supplied regarding the registration of a newly described species: Publication LSID: urn:lsid:zoobank.org:pub:6DA69051-BDCF-4439-9F55-121F817D2348 *Westheideius*: urn:lsid:zoobank.org:act:1EF85063-0750-478A-814C-44E0E45A2DFA *Struwela camposi*: urn:lsid:zoobank.org:act:76CAF1ED-BEBE-44E6-B2E1-3045D42C4DC4
